# Multilabel multiclass sentiment and emotion dataset from indonesian mobile application review

**DOI:** 10.1016/j.dib.2023.109576

**Published:** 2023-09-15

**Authors:** Riccosan, Karen Etania Saputra

**Affiliations:** Computer Science Department, School of Computer Science, Bina Nusantara University Bandung Campus, Jakarta, Indonesia 11480

**Keywords:** Sentiment, Emotion, Text classification, Dataset, Multilabel, Multiclass, Mobile application review, Indonesia

## Abstract

Reviews are a person's way of expressing feedback on something in the form of criticism and ideas. Reviews of mobile apps are a type of user feedback that focuses on the performance and look of a mobile application and is typically featured on the download page of a mobile application, such as in the Apps Store. Because it comprises a person's feelings and emotions, whether they are joyful, sad, hostile, or indifferent toward a mobile application, the review data is textual and may be gathered and utilized as material for creating a textual dataset. This work creates a multi-label multi-class Indonesian-language dataset based on public reviews of mobile applications with sentiment and emotional values. Another factor supporting the creation of this dataset is the fact that there is still a limited number of textual datasets based on the Indonesian language that are multi-label multiclass for performing sentiment analysis tasks, particularly those linked to text classification tasks. The data generated by this research was cleaned and handled during the pre-processing step and was annotated with 3 sentiments, namely positive, negative, and neutral, as well as 6 emotions, namely anger, fear, sad, happy, love, and neutral.

Specifications Table

**Table:** 

Subject	Data Science, Applied Machine Learning, Data Mining and Statistical Analysis
Specific subject area	This dataset was created to carry out sentence classification tasks in Indonesian language as part of Natural Language Processing utilizing multiclass multilabel data for sentiment analysis. This dataset is multiclass because it is annotated with 3 sentiment labels (positive, negative, and neutral) and 6 emotion labels (sad, fear, anger, happy, love, and neutral), and multilabel because each sentence containing both labels, a sentiment and an emotion label.
Data format	Raw, Filtered, Annotated
Type of data	Text, Table
Data collection	The data was gathered through a scraping procedure of mobile app reviews in Indonesia using Google Colab and the TQDM package for the Python programming language. Several Mobile Application IDs obtained from the Link to download the corresponding application are used to carry out the data search. Furthermore, the search process is filtered using search criteria that involve lang = 'Id' and country = 'Id' to acquire particular data originating in Indonesia and only in Indonesian language.
Data source location	Indonesia
Data accessibility	Repository Name: **Zenodo**. Direct URL to Repository: https://doi.org/10.5281/zenodo.8268213 Instructions for accessing these dataset (1-5) or source code (6-8): 1. Access the link provided above this instructions. 2. Download directly the .Zip file for the entire dataset. 3. Extract the data to local folder. 4. Go into a folder with the following name to access **Dataset**: “**Multilabel Sentiment and Emotion Dataset from Indonesian Mobile Application Review**” 5. The data file can be opened with .txt file reader or tabular data reader (i.e. Ms. Excel), or open it in another IDE (i.e. G-Colab or Jupyter Nb.). 6. Go into a folder with the following name to access source code: “**Data Scrapping Code**” 7. Inside the folder is a .ipynb (Python Notebook) file for the source code. 8. Open the Python Notebook file with Google Colab or Jupiter Notebook.

## 1 Value of the Data

• Contains review text with Indonesian language for mobile applications in Indonesia.
• The data is intended to aid research in the field of Indonesian sentiment analysis as part of the basic Natural Language Processing task of text classification, specifically for the sentiment classification or the emotion classification, or a combination of the two basic tasks.
• This dataset can be utilized as primary or secondary data for sentiment or emotion classification tasks, or a mix of the two basic tasks.
• This data may be utilized to help text classification jobs in the form of emotions and sentiments in combination with different languages with machine learning or deep learning architectures.
• This Mobile App Review dataset can support researchers and organizations to gather and classify sentiments and emotions from Indonesian citizens about mobile apps in order to better understand what characteristics Indonesians want from mobile applications.
• Given the enormous number of mobile application users in Indonesia, a dataset for sentiment analysis research is required, particularly for identifying the sentiments and emotions of Indonesian peoples toward mobile apps.
• There is still not a lot of multi-label multi-class data in Indonesian for the task of sentiment classification, emotion classification, or a combination of the two.

## 2 Data Description

**Fig. 1:**
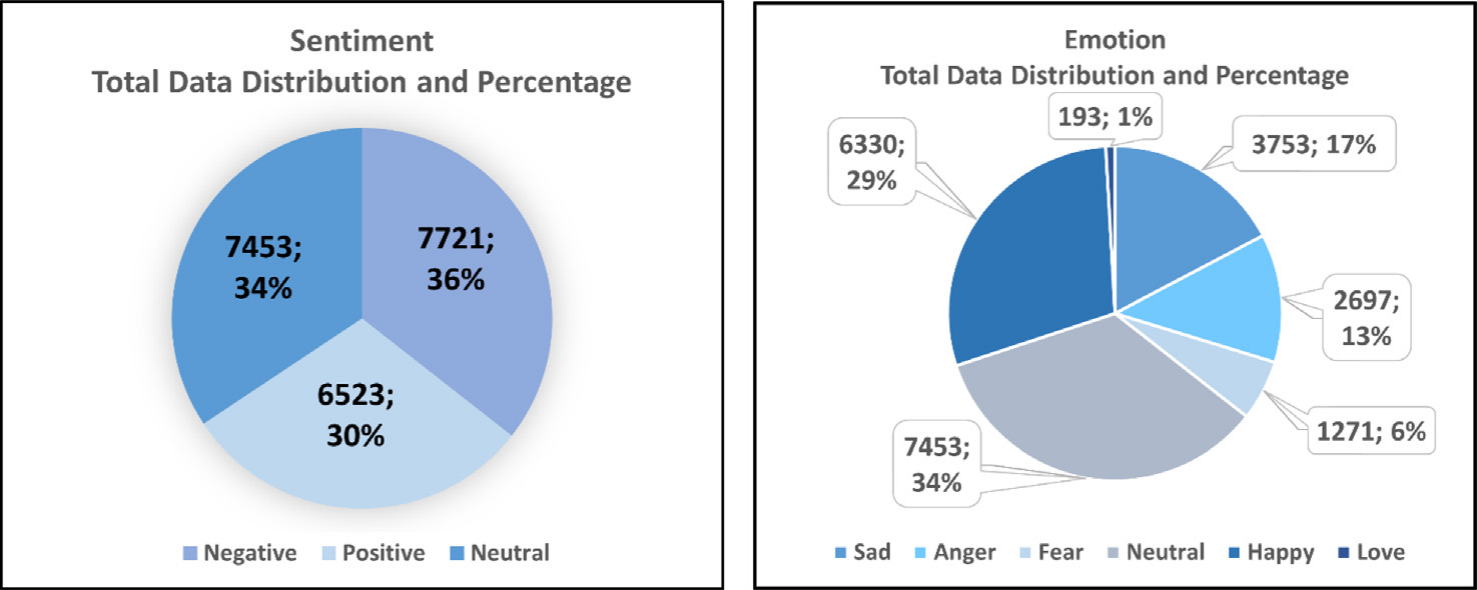
Sentiment & emotion total data distribution and percentage base on classes.

The research's dataset was assembled from indonesian public reviews of mobile applications in Indonesia. This dataset consists of sentences with multi-label properties because they contain a sentiment label and an emotion label, and it is multi-class since it has 3 sentiment classes (positive, negative, neutral) and 6 emotion classes (anger, sad, fear, happy, love, neutral). The total data formed until the end of the research amounted to **21,697 data** of Indonesian sentences and has been annotated by dividing data for Sentiment Data in the form of **7,721 Negative Data, 36%** from total data which is broken down into 3 Emotion Classes, namely 3,753 Sad, 2,697 Anger, and 1,271 Fear; **6,523 Positive Data, 30%** from total data which is broken down into 2 Emotion Classes, namely 6,330 Happy and 193 Love; and **7,453 Neutral Data, 34%** from total data which contain Neutral Emotion Class. The amount of data constructed varies by class, as shown in Table 1 and the image in Fig. 1 for information on the percentage of data distribution for each sentiment and emotion class.

**Table 1:** Final data distribution base on sentiment & emotion class after pre-processing stage.

Sentiment Class	Negative			Positive		Neutral
Emotion Class	Sad	Anger	Fear	Happy	Love	Neutral
Total Data	3,753	2,697	1,271	6,330	193	7,453

This research's final output is a dataset that saved in the form of a ".csv file." The data in the file is divided into three columns: content, sentiment, and emotion. The content column includes data in the form of Indonesian sentences consisting of user reviews for the mobile app. Meanwhile, the sentiment and emotion columns for associated data hold a single label from the sentiment and emotion classes, respectively. For an example, please look at Table 2. The dataset file may be accessed using the URL provided in the Specification Table, inside the Data accessibility section.

**Table 2:** Data example and column distribution inside CSV file.

content	Sentiment	Emotion
[***example data in Indonesian***] cinta mati deh ama game ini maknyus	Positive	Love
[***example data in Indonesian***] Semenjak update malah ada bug meresahkan seperti ngefreze saat perjalanan, jadi gak ke save padahal dah jauh	Negative	Anger
[***example data in English for review***] love to death with this game great	Positive	Love
[***example data in English for review***] Since the update, there have been troubling bugs like freezing while traveling, so it doesn't save even though it's far away	Negative	Anger

## 3 Experimental Design, Materials and Methods

**Fig. 2:**
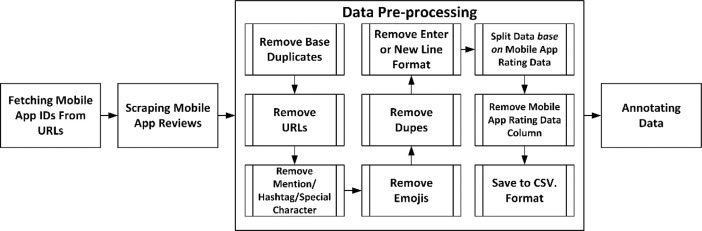
Research method.

The applied research flow has 4 major stages consisting of *fetching mobile app ID's from URLs, scraping mobile app reviews, data pre-processing*, and *annotating data* as shown in Fig. 2. In step 1, searching process for mobile applications was conducted for obtaining public reviews. For this study, 10 mobile applications in Indonesia were employed, and the names of the applications used for data scraping are not mentioned in this research report in order to preserve the anonymity and confidentiality of firm data that has related applications. The following step collects data in the form of Application IDs derived from the URL used to download the corresponding application. After obtaining the Application IDs, we check the information from the mobile application via the Application ID in Google Colab using the “Tqdm Package” for the Python programming language to ensure several requirements before beginning the data scraping process, such as large number of reviews, the application being in the Indonesian region, and using Indonesian language.

For the next step, *scrapping mobile app reviews*, the “Tqdm Package” is utilized again for the Python programming language to collect mobile app review data. Several data search parameters are used, including *min_data* = 25,000 (the aggregated minimum total data from all mobile applications), *lang* = ‘id', *country* = ‘id', and *filter_score* ranging from 1 to 5. The *lang* and *country* parameters ensure that data originates from Indonesian mobile applications and that mobile app evaluations are expressed in Indonesian. The *filter_score* parameter was used in the process to guarantee that any collected mobile app review includes a rating for the application. Raw data for this research was collected from 10 mobile applications, totaling of 37,108 mobile app reviews using the data scraping procedure.

The third step is *data pre-processing*, which is made up of 9 sub-processes (check Fig. 2). The basic data pre-processing procedure is employed at this step to remove unnecessary data [1], in the form of *remove base duplicates, remove URLs, remove mention/hashtag/special character, remove emoji, removes dupes*, and *remove enter/new line format*. All of the basic data pre-processing used is a stage known as Cleaning Text [2]. However, several processes are not implemented, such as punctuation removal and stop-word removal in order to preserve the cultural aspect in understanding the sentiment and emotion value contained in the data, and word separation because, with the exception of slank words, there is almost no combining of words to make them shorter in Indonesian sentences.

After the data has been cleaned, *split data based on mobile app rating level* is applied and from that process the data divided into 5 dataframes base on user rating level on mobile app. The column carrying mobile app rating level was removed after the data was separated since the data created via this research only intended to take pure textual data. For the last step in *data pre-processing*, every dataframes saved into ".CSV File" format. To access the source code for the data scrapping process [3], please access this link provided in the **Specification Table – Data accessibility**.

*Annotating data* is the final step of creating this dataset, and its performed by two annotators, who are natives and utilizing Indonesian as the mother tongue. Because process of annotating data in the form of sentences used in the creation of this dataset is one of the processes involved in identifying a kind of sentiment or emotion included in a sentence, it is also known as the Sentiment Analysis process [4] and this approach is also a type of text analysis since it attempts to extract some values (sentiment and emotion) from Indonesian sentences [5]. For the data annotation with six emotion labels, in the form of five basic human emotions and neutral class, based on phrases containing values from the Emotion Hierarchy Level theory [6] which is translated into Indonesian (original keyword data displayed on Table 3 and Indonesian language keyword data is displayed on Table 4), and Shaver's theory of basic human emotions [7] which was later popularized as Parrot's Basic Emotion [8] which is used as a supporting theory for the annotator to determines the type of emotion class in the data.

**Table 3:** Emotion hierarchy in English [6].

Emotion	Subordinate
Love	(1) adoration, affection, love, fondness, liking, attraction, caring, tenderness, compassion, sentimentality; (2) arousal, desire, lust, passion, infatuation; (3) longing
Happiness	(1) amusement, bliss, cheerfulness, gaiety, glee, jolliness, joviality, joy, delight, enjoyment, gladness, happiness, jubilation, elation, satisfaction, ecstacy, euphoria; (2) enthusiasm, zeal, zest, excitement, thrill, exhilaration; (3) contentment, pleasure, pride, triumph; (4) eagerness, hope, optimism; (5) enthrallment, rapture; (6) relief
Anger	(1) aggravation, irritation, agitation, annoyance, grouchiness, grumpiness; (2) exasperation, frustration; (3) anger, rage, outrage, fury, wrath, hostility, ferocity, bitterness, hate, loathing, scorn, spite, vengefulness, dislike, resentment; (4) disgust, revulsion, contempt; (5) envy, jealousy; (6) torment
Fear	(1) alarm, shock, fear, fright, horror, terror, panic, hysteria, mortification; (2) anxiety, nervousness, tenseness, uneasiness, apprehension, worry, distress, dread
Sadness	(1) agony, suffering, hurt, anguish; (2) depression, despair, hopelessness, gloom, glumness, sadness, unhappiness, grief, sorrow, woe, misery, melancholy; (3) dismay, disappointment, displeasure; (4) guilt, shame, regret, remorse, alienation, isolation, neglect, loneliness, rejection, homesickness, defeat, rejection, insecurity, embarrassment, humiliation, insult; (5) pity, sympathy

**Table 4:** Emotion hierarchy in English [6] translated into Indonesian language.

Emotion	Subordinate
Love	(1) pujaan, kasih, cinta, kegemaran, kesukaan, daya tarik, kepedulian, kelembutan, belas kasih, sentimental; (2) gairah, hasrat, nafsu, semangat, jatuh hati; (3) rindu
Happiness	(1) hiburan, kebahagiaan, kegembiraan, keriangan, keriaan, gaul, sukacita, menyenangkan, kenikmatan, sorak bahagia, kegirangan hati, kepuasan, luar biasa gembira, euforia; (2) antusiasme, semangat, gairah semangat, ketertarikan, sensasi; (3) kepuasan, kelezatan, kebanggaan, kemenangan; (4) keinginan, harapan, optimisme; (5) pesona, girang melayang; (6) kelegaan
Anger	(1) jengkel, iritasi, agitasi, ketergangguan, kekesalan, amarah; (2) kejengkelan, frustasi; (3) marah, gusar, kebiadaban, murka, sangat murka, kebencian, keganasan, kepahitan, benci, kebencian, cemooh, dengki, dendam, tidak suka, kedendaman; (4) jijik, muak, penghinaan; (5) iri, cemburu; (6) siksaan
Fear	(1) alarm, terkejut, takut, ngeri, horor, teror, panic, histeris, malu; (2) kecemasan, kegugupan, ketegangan, gelisah, prihatin, khawatir, kesulitan, ketakutan
Sadness	(1) penderitaan, sengsara, sakit, derita; (2) depresi, putus asa, keputusasaan, suram, kesuraman, kesedihan, ketidakbahagiaan, duka, nestapa, duka cita, melarat, melankolis; (3) cemas, kecewa, ketidaksenangan; (4) kesalahan, rasa malu, sesal, rasa bersalah, pengasingan, isolasi, ketelantaran, kesendirian, penolakan, kerinduan, kekalahan, penolakan, ketidakamanan, kejengahan, penghinaan, penistaan; (5) belas, simpati

Therefore, the annotator uses the reference value for the word content supplied in Table 4 throughout the annotation process when applying labels from emotion classes. For the process of annotating data that does not contain one or more words from Table 4, it will use the process of interpreting the data in the form of an entire sentence as a whole data, then processing whether the sentence contains one of the meanings that contain the nature of words that contain emotional value according to the list of words in Table 4. Specifically, the Neutral Emotion class was applied to data in the form of *announcements, news, notifications sentence, random letters* (i.e. “awdj!! #zv^v%s kwdn!@r goodrt”, the data annotated with neutral emotion class) or data that does not contain words about five basic emotions in Indonesian Language as shown in Table 4. For data annotations utilizing the sentiment class is organized by emotion class, in the form of the Positive class, with 2 emotion: Happy and Love, the Negative class, with 3 emotion: Anger, Sad, and Fear, and the Neutral class, which contains the Neutral Emotion class. For example, if there is data with the emotion label Fear or Sad or Anger, use the Negative class for the sentiment label annotation (i.e. “I feel so broken after playing that @#fes! game and messed up, arghhhhhhh rampageee”, Emotion: **Anger**, Sentiment: **Negative**).

After the data annotation process finished by all annotators, the Inter-annotator Agreement (IAA) value is calculated, which is the value of the amount of annotation similarity between the annotator subjects. The IAA is calculated using the Kappa's Statistics approach [9] in the form of Cohen's Kappa, which is broken into several sections. *First* **{1}**, calculate the Cohen's Kappa value individually for the two types of classes, sentiment and emotion. *Second* **{2}**, the average Cohen's Kappa calculation in the form of the Cohen's Kappa value for the sentiment class is added to the Cohen's Kappa value for the emotion class and divided by 2 type of class categories (sentiment and emotion). *Third* **{3}**, the Cohen's Kappa calculation is performed by integrating feelings and emotions into a single label with comma separator. Table 5 displays the results of calculating the IAA values with Cohen's Kappa, and it is known that the average Inter-annotator agreement value for two annotator subjects is in the range of **61.14%**, indicating that the IAA level of the dataset from this research is in **the Moderate Level of Aggrement**.

**Table 5:** Inter-annotator agreement value with Cohen's Kappa method.

Cohen's Kappa Method	Kappa Value
**{1}** Sentiment Class	0.6252
**{1}** Emotion Class	0.6044
**{2}** Mean of Sentiment Class + Emotion Class	0.6148
**{3}** Merged Sentiment & Emotion Class	0.6013
**Average Cohen's Kappa Value**	**0.6114**
**Percentage Final Cohen's Kappa Value**	0.6114 × 100% = **61.14%**

## 3 Limitations

This dataset's development is hampered by limitations that emerge during data pre-processing. Even though the data was already searched using language and geographical features, the data contained additional languages and the existence of random letters, necessitating manual cleaning and translation during data annotation.

## Ethics Statement

All data in this research was gathered by the authors. The data used to build the dataset in this research has been totally anonymized (user ID, username, mobile app developer, app name, and app ID) for security and confidentiality, as well as to comply with data redistribution regulations. According to Google Play's Terms of Service (ToS), permission is obtained to download, view, or use content available for free on the Google PlayStore, but there are some limitations that must be applied, particularly in this case about the mobile app review, where all form of content relating to user or the app developer personal information must be disguised or deleted in order to support confidentiality and data security, and all content collected for this research is purely for research purposes, not-for-profit, and may not be traded in any form. The authors in this article have read and follow the ethical requirements for publication in Data in Brief and confirming that the current work does not involve human subjects, animal experiments, or any data collected from social media platforms.

## CRediT authorship contribution statement

**:** Conceptualization, Methodology, Writing – original draft, Writing – review & editing, Validation, Data curation. **Karen Etania Saputra:** Data curation, Validation, Writing – review & editing.

## Data Availability

Multilabel Multiclass Sentiment and Emotion Dataset from Indonesian Mobile Application Review (Original data) (Zenodo)
